# Maternal plasma and salivary anelloviruses in pregnancy and preterm birth

**DOI:** 10.3389/fmed.2023.1191938

**Published:** 2023-06-15

**Authors:** Chandrashekara Kyathanahalli, Madeline Snedden, Lavisha Singh, Camilla Regalia, Lauren Keenan-Devlin, Ann E. Borders, Emmet Hirsch

**Affiliations:** ^1^Department of Obstetrics and Gynecology, NorthShore University HealthSystem, Evanston, IL, United States; ^2^Department of Obstetrics and Gynecology, Pritzker School of Medicine, University of Chicago, Chicago, IL, United States; ^3^Department of Statistics, NorthShore University HealthSystem, Evanston, IL, United States; ^4^Center for Healthcare Studies, Institute for Public Health and Medicine, University of Chicago Pritzker School of Medicine, Northwestern University, Evanston, IL, United States

**Keywords:** human anellovirus, torque teno virus, torque teno mini virus, pregnancy, maternal blood and saliva, term and preterm birth

## Abstract

**Introduction:**

Human anelloviruses, including torque teno virus (TTV) and torque teno mini virus (TTMV), are ubiquitous in the general population and have no known pathogenicity. We investigated the prevalence and viral load of TTV and TTMV in plasma and saliva over pregnancy, and assessed their association with spontaneous or medically indicated preterm birth.

**Methods:**

This is a secondary analysis of the Measurement of Maternal Stress (MOMS) study, which recruited 744 individuals with singleton pregnancies from 4 US sites (Chicago, Pittsburgh, San Antonio, and rural Pennsylvania). Baseline outpatient visits took place in the second trimester (between 12′0 and 20′6/7  weeks’ gestation), and follow-up visits in the third trimester (between 32′0 and 35′6/7  weeks’ gestation). In a case-control study design, participants who delivered preterm (<37 weeks) resulting from spontaneous labor and/or preterm premature rupture of membranes (“sPTB”) were compared with participants experiencing medically indicated preterm birth (“iPTB”), or delivery at term (“controls”). Plasma and saliva samples obtained during the second and third trimesters were tested for the presence and quantity of TTV and TTMV using real-time PCR. Demographic data were obtained via self-report, and clinical data via medical record review by trained research personnel.

**Results:**

TTV was detected in plasma from 81% (second trimester) and 77% (third trimester) of participants, and in saliva from 64 and 60%. Corresponding detection rates for TTMV were 59 and 41% in plasma, and 35 and 24% in saliva. TTV and TTMV concentrations were similar between matched plasma and saliva samples. TTV prevalence and concentrations were not significantly different between groups (sPTB, iPTB, and controls). However, plasma TTMV in the third trimester was associated with sPTB and earlier gestational age at delivery. The iPTB group was not different from either the sPTB or the control group. In saliva, concentrations of TTV and TTMV were similar among the three groups. Both TTV and TTMV were more prevalent with increasing parity and were more common in Black and Hispanic participants compared to non-Hispanic White participants.

**Conclusion:**

Anellovirus presence (specifically, TTMV) in the third trimester may be associated with preterm birth. Whether this association is causative remains to be determined.

## 1. Introduction

Preterm birth (PTB) is the most common cause of neonatal mortality and a major contributor to long-term adverse health outcomes (1). As PTB is a heterogeneous condition with multiple causes, its pathogenesis remains poorly understood. PTB may result from spontaneous preterm labor with intact fetal membranes or from preterm premature rupture of the fetal membranes (PPROM), collectively referred to in this paper as “spontaneous preterm birth” (sPTB). Alternatively, some preterm births are the result of medical intervention due to a variety of maternal or fetal indications (referred to in this paper as “indicated PTB,” or iPTB) (2). A significant proportion of PTB is associated with microbial invasion of the gestational compartment, with the highest likelihood of microbial invasion in sPTB with extreme prematurity (i.e., before 28 weeks’ gestation) (3–5). Nonetheless, the role of microbes in the absence of overt infection remains elusive (6). This is at least in part because infection may be subclinical, and because in most instances it is not clear if colonization of the gestational compartment is a cause or a consequence of labor. Furthermore, traditional bacterial culture techniques are limited compared to molecular diagnosis (4, 7). The potential involvement of viruses in the pathogenesis of PTB has been underexplored. As a result, there is limited knowledge of normal and pathogenic microbiota and their relationship to labor.

Epidemiological studies have demonstrated an association between acute viral infections (such as influenza (8), HIV (9, 10), hepatitis B (11)), and PTB (8, 9, 12–14). Although the mechanisms are unclear, there is evidence that viral infection may predispose the pregnancy to preterm labor and preterm delivery by augmenting background inflammation and sensitizing the individual to secondary bacterial infections (15).

Mounting evidence indicates that some viruses may engage in benign colonization of the human body without causing clinical symptoms or disease (16). Among such microbes are anelloviruses: highly prevalent, small, non-enveloped, single-stranded circular DNA viruses (17). Humans are nearly always colonized with at least 1 of 3 anellovirus subtypes, namely *Alphatorquevirus* (comprising species of torque teno virus, TTV), *Betatorquevirus* (torque teno mini virus, TTMV), and *Gammatorquevirus* (torque teno midi virus, TTMDV) (18). In healthy pregnant individuals, the prototypical anellovirus, TTV, has been found in blood (19), amniotic fluid (20), cervical (21) and vaginal secretions (22), breast milk (20), and saliva (23). Thus far, anelloviruses have not been linked definitively to any active disease states, but higher viral loads are associated with unexplained fever (24), immune suppression (25), bronchopneumonia (26), and cancer (27).

We previously identified a higher prevalence of anelloviruses (specifically, either TTV or TTMV) in the serum of laboring patients who spontaneously delivered preterm compared to those who delivered at term, suggesting that these viruses may have a role in determining parturition timing (28). Though that study uncovered a previously unappreciated association of anelloviruses with the timing of spontaneous labor, the findings were qualitative and derived from a single patient population. Furthermore, in the prior study, all samples were collected from pregnant persons in labor.

In the present study, we determined the plasma and salivary loads of TTV and TTMV at two points during pregnancy (second and third trimesters) in a subpopulation derived from a large prospective multicenter observational cohort study (the MOMS study (29)). The prevalence and copy numbers of TTV and TTMV in plasma and saliva were studied with respect to stage of pregnancy and pregnancy outcomes.

## 2. Materials and methods

### 2.1. Participants and study sites

The current study was conducted in a subset of participants enrolled in the multisite Measurement of Maternal Stress (MOMS) study (29). MOMS recruited 744 individuals with singleton pregnancies from antenatal clinics in Chicago, IL; Pittsburgh, PA; Schuylkill County, PA; and San Antonio, TX, between June 2013 and May 2014. Eligible participants were 18 years or older, and less than 21 weeks’ gestation at enrollment. Participants were not eligible if there was known fetal congenital anomaly, chromosomal abnormalities, progesterone treatment after 14 weeks’ gestation, or chronic corticosteroid treatment. Informed consent was obtained from all participants, and the study protocol was approved by the Institutional Review Board (IRB) of Northwestern University as well as the IRBs of all participating sites.

Maternal race and ethnicity, age, parity, prior PTB, and smoking history were self-reported. Clinical variables, including gestational age, PTB in the index pregnancy, and reason for delivery, were abstracted from electronic medical records.

Baseline visits and specimen collection took place once in the second trimester (12–20′6/7 weeks’ gestation), and again in the third trimester (32–35′6/7 weeks’ gestation). Plasma was harvested within 30 min of collection by centrifuging whole blood obtained via antecubital venipuncture into 10 mL EDTA-coated Vacutainer tubes (BD Biosciences) at 2,000 × g at 4°C for 15 min, and then aliquoted. Participants were asked to refrain from eating or chewing gum for at least 1 h before saliva collection. Saliva was collected without any stimulation by having participants hold a cotton swab in their mouth without chewing for 3 min. The swabs were then centrifuged at 1,000 × g for 2 min at room temperature and discarded. Plasma and saliva samples were frozen at −80°C until assay.

**Figure 1:**
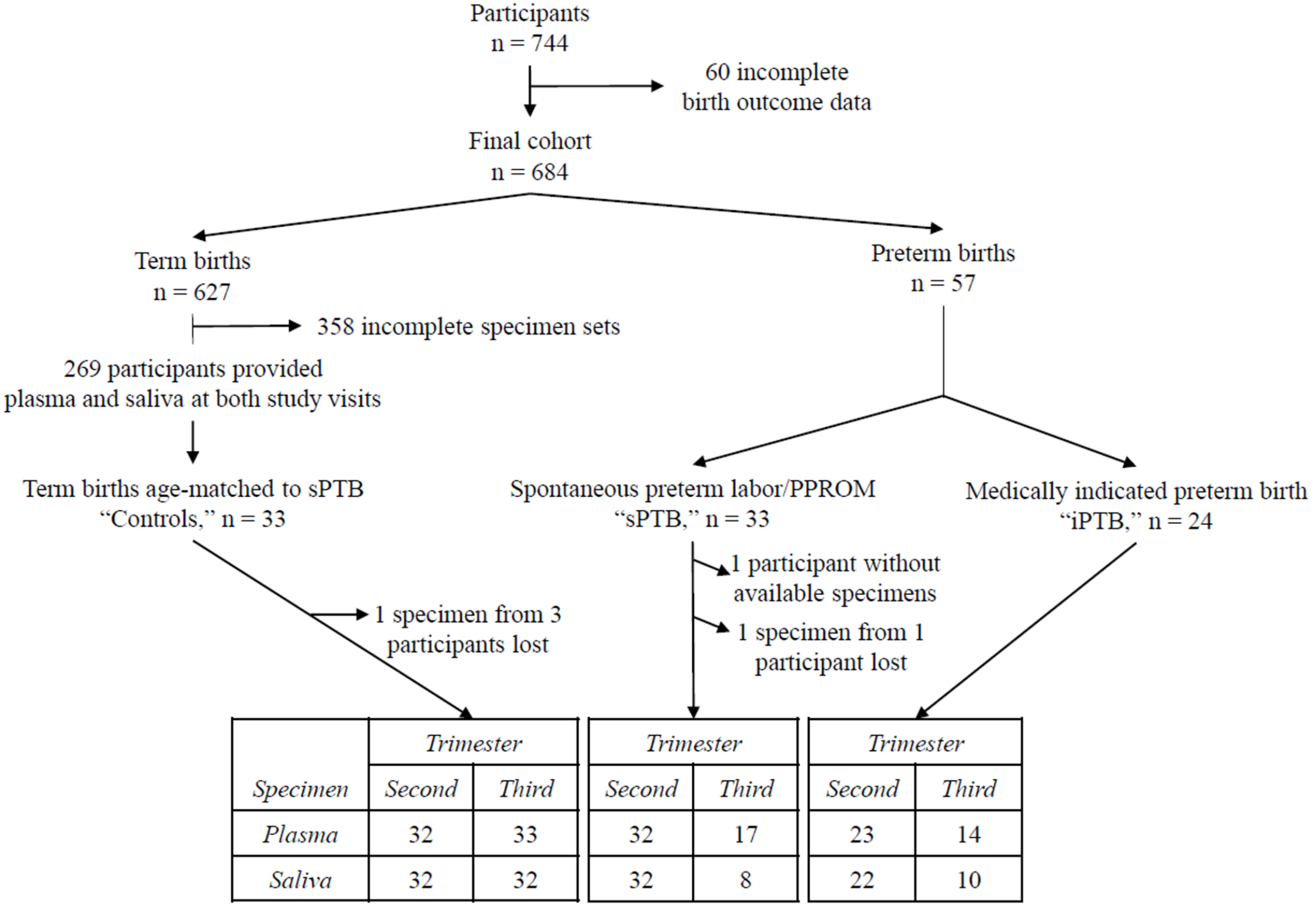
Flowchart of the study population. Seven hundred and forty-four participants were recruited in the original MOMS study (29). Baseline visits took place between 12′0 and 20′6/7 weeks’ gestation (second trimester), and follow-up visits between 32′0 and 35′6/7 weeks’ gestation (third trimester). Sixty participants were excluded for incomplete birth outcomes, leaving 684 in the final study cohort. Six hundred and twenty-seven of these had term deliveries (≥37′0 weeks’ gestation). Fifty seven delivered prematurely, 33 after a spontaneous labor event (“sPTB”) and 24 due to a medical indication (“iPTB”). All participants delivering preterm were included in the present study. Of those who delivered at term, matched plasma and saliva samples were available from both study visits in 269 participants. “Control” participants were selected from this subset, matched to sPTB by maternal age. The table at the bottom of the figure provides a summary of the blood and saliva samples from each birth outcome group used for this study.

For this report, we used archived frozen plasma and saliva collected from 3 groups of pregnant participants according to the timing and instigating event for delivery: (a) all participants who delivered preterm (<37 weeks’ gestation) as a result of spontaneous labor or PPROM (“sPTB,” *n* = 32); (b) all participants whose preterm delivery was initiated for medical reasons (“iPTB,” *n* = 24); and (c) controls (age-matched to the sPTB group) who delivered at term (between 37–41′6/7 weeks’ gestation; “controls,” *n* = 33). The flowchart in Figure 1 depicts participant selection and biospecimens used for this study.

The following samples are missing from the set: 11 participants delivered before 32 weeks’ gestation (the minimum gestational age for the third trimester study visit); 2 participants failed to attend the second study visit. Seventeen participants completed their second study visit, but either plasma or saliva samples were not available. Four samples were lost during nucleic acid extraction. Overall, 45 participants had complete matched sets of extracted viral nucleic acids: 30 control, 8 sPTB, and 7 iPTB participants.

### 2.2. Isolation of viral nucleic acids from plasma and saliva samples

Viral nucleic acids were extracted from 200 μL plasma or saliva using a QIAamp MinElute Virus Spin Kit (Qiagen, Valencia, CA) following the manufacturer’s instructions and eluted into 65 μL of nuclease-free water (Invitrogen, Carlsbad, CA). The efficiency of DNA extraction and the absence of PCR inhibitors were confirmed by “spiking” the samples with QuantiFast Internal Control DNA (a synthetic DNA oligonucleotide whose sequence is not naturally occurring, Qiagen), according to the manufacturer’s protocol.

### 2.3. Anellovirus PCR analysis

#### 2.3.1. Construction of TTV and TTMV standard curves

A 63 bp region of the TTV genome (5′-GTGCCGAAGGTGAGTTTACACACCGAAGTCAAGGG GCAATTCGGGCTCGGGACTGGCCGGGCT-3′, GenBank accession number AB008394) and a 96 bp sequence of the TTMV genome (5′-AGTTTATCACGCCAGACGGAGACGGCATCAGAACACTGACTGCCGGCTGATCTTGGGCGGGAGCCGAAGGTGAGTGAAACCACCGAAGTCTAGGGG-3′, GenBank accession number AB026930) were synthesized (IDT, Coralville, IA) and used to generate standard curves for quantitation. A linearity study was performed on serial dilutions of the oligonucleotides in nuclease-free water using TTV- and TTMV-specific primers and probes as described below. At each concentration, up to 8 replicates were tested on at least 2 separate days. Assay variability was evaluated by calculating coefficients of variation (CVs) comparing the threshold cycles (Cqs) from the same run (intra-assay) and from different days (inter-assay). A line of best fit was applied with regression analysis on a plot of the average Cq (y-axis) and log oligonucleotide concentration (x-axis).

**Figure 2:**
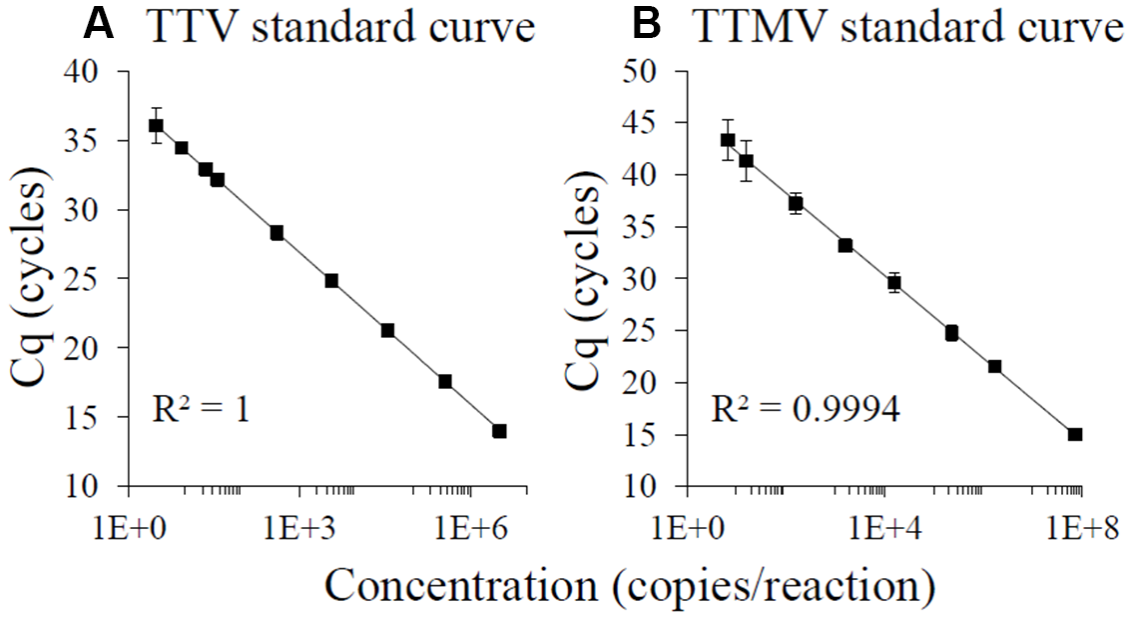
TaqMan-based standard curves of TTV and TTMV oligonucleotides. 100-, 10-, and 2-fold serial dilutions ranging from approximately 10^8^ to 10^0^ copies of TTV **(A)** or TTMV **(B)** oligonucleotides per reaction were amplified using real-time PCR, with at least 4 replicates per concentration. A line of best fit was regressed over the average Cq from the highest log concentration with all Cqs ≥12 cycles to the lowest log concentration at which all replicates produced detectable Cqs and had a coefficient of variation <35%. Assay characteristics: TTV: slope = −3.60, intercept = 37.76, *R*^2^ = 1.0000, 89% efficiency; TTMV: slope = −3.81, intercept = 45.00, *R*^2^ = 0.9994, 83% efficiency. Error bars = 1 standard deviation.

Each assay had intra-assay CVs below 5% and inter-assay CVs below 10%. Standard regression analyses of the linear parts of the curves (for TTV: 3 × 10^0^ to 3 × 10^6^ TTV copies/reaction, LOQ equivalent to 3 × 10^2^ TTV genomes/mL; for TTMV: 7 × 10^0^ to 7 × 10^7^ TTMV copies/reaction, LOQ equivalent to 4 × 10^2^ TTMV genomes/mL) produced best fit lines with the following characteristics: TTV: slope = −3.60, intercept = 37.76, *R*^2^ = 1.0000, 89% efficiency (Figure 2A); TTMV: slope = −3.81, intercept = 45.00, *R*^2^ = 0.9994, 83% efficiency (Figure 2B).

#### 2.3.2. TTV and TTMV DNA quantification in plasma and saliva samples

TaqMan quantitative real-time PCR was performed in duplicate to measure TTV and TTMV DNA in plasma and saliva samples using primers specific to each genus. Primers and probes were purchased from Sigma-Aldrich (St. Louis, MO).

The TTV assay targeted a highly conserved segment of the viral untranslated region upstream of open reading frame (ORF) 1 (26) and was performed in 20 μL total volume, including 3 μL viral nucleic acids extracted from plasma or saliva, TaqMan Fast Advanced Master Mix (Thermofisher), 0.9 μM of forward (5′-GTGCCGIAGGTGAGTTTA-3′) and reverse primers (5′- AGCCCGGCCAGTCC-3′), and 250 nM of probe (5′-TCAAGGGGCAATTCGGGCT-3′, 6-FAM/BHQ1). Cycling conditions were 50°C for 2 min and 95°C for 20 s, followed by 40 cycles of 95°C for 3 s and 60°C for 30 s.

The TTMV assay targeted a short segment near ORF2 (30) and was performed in 20 μL total volume, including 6 μL viral nucleic acids extracted from plasma or saliva, TaqMan Fast Advanced Master Mix, 1.0 μM of forward (5′-AGTTTATGCCGCCAGACG-3′) and reverse primers (5′-CCCTAGACTTCGGTGGTTTC-3′), and 250 nM probe (5′-ACTCACCTHCGGCACCCG-3′, 6-FAM/TAMRA). Cycling conditions were 95°C for 20 s, followed by 50 cycles of 95°C for 1 s and 60°C for 20 s. The reduced amplification efficiency of 83% for the TTMV amplicon is due to tetranucleotide content (i.e., 4 Gs in tandem) and accounts for the need for both a larger quantity of template and a greater number of amplification cycles (as many as 50). These characteristics are consistent with multiple reports in the literature (31–34).

To assess for false positives (i.e., amplification resulting from a minute quantity of contaminants), two types of no-template control were employed. (1) Nuclease-free water was used as the starting material in place of saliva or plasma. The resulting nucleic acid extracts were assayed for TTV or TTMV on the same PCR plates as the biological samples extracted that day. (2) On every PCR assay plate, replicate wells were supplied with nuclease-free water in place of extracted nucleic acids. Thirty such negative controls were run on 11 96-well PCR plates over the course of the experiments described. None had false-positive amplification.

All PCR reactions were performed and analyzed on a StepOnePlusTM Real-Time PCR System (Applied Biosystems). Viral DNA was classified as “present” if its quantity was above the limit of detection (LOD, TTV Cqs <40 cycles or TTMV Cqs <50 cycles), and “absent” if quantity was below the LOD, an approach that follows published methods for anellovirus PCR detection (24, 35–39). Quantitative viral loads were determined using the standard curves described above. Values below the limit of quantitation (LOQ) were handled according to published procedures (40–43). Such samples were segregated into 3 tiers of descending nucleic acid quantity: (a) detectable but not quantifiable (two positive PCR assays below the LOQ); (b) at the limit of detection (one detectable and one non-detectable assay); and (c) non-detectable.

### 2.4. Statistical analyses

Participant demographic and clinical characteristics were compared between birth outcome groups (controls, sPTB, iPTB) as follows: Continuous variables, such as maternal age or gestational age, were compared using the non-parametric Kruskal-Wallis test followed by Dwass, Steel, Critchlow-Fligner (DSCF) test for pairwise comparison. Categorical variables, such as presence/absence of TTV and TTMV, BMI category or smoking history, were compared using Chi-squared or Fisher’s exact test, followed by pairwise comparison with Bonferroni correction as appropriate. Concentrations of TTV and TTMV (i.e., continuous variables, including the assigned values described above for measurements below the LOQ) were compared using non-parametric methods: Wilcoxon rank sum test (for 2 groups) and Kruskal-Wallis test (>2 groups) followed by DSCF test. Gestational age at delivery between virus-positive and virus-negative groups was compared using Wilcoxon rank sum test. The association between virus concentration and gestational age at delivery was determined using Spearman’s rank correlation test. Generalized mixed linear models were used to determine associations between virus concentrations in plasma vs. saliva collected from the same study visit and the association between virus concentrations in the second vs. third trimesters. To test the difference in virus concentration between trimesters within a birth outcome group, multiple comparisons with Tukey adjustment were performed. The difference of least square mean estimates with adjusted 95% confidence intervals and *p*-values are presented. *p*-values < 0.05 were considered statistically significant. All statistical analyses were performed using SAS version 9.4 (SAS Institute Inc., Cary, NC, United States).

## 3. Results

### 3.1. Demographic and clinical characteristics of the study participants

A flow chart of participants selected from the original MOMS cohort included in this study is presented in Figure 1. From the original cohort (*n* = 744), full birth outcome data were available in 684 participants. Among these, 57 delivered preterm (before 37 weeks’ gestation), a prevalence rate of 8.3%. Of those who delivered preterm, 58% of births resulted from either spontaneous preterm labor or PPROM (“sPTB,” *n* = 33), and 42% were medically indicated preterm births (“iPTB,” *n* = 24). Thirty-three people who delivered at term (37–41′6/7 weeks’ gestation) and had available plasma and saliva specimens from both study visits were age-matched to the sPTB cases and selected as controls.

Demographic and clinical characteristics of individuals included in this study are presented in Table 1. Except for gestational age at delivery and race/ethnicity, there were no statistically significant differences between the groups.

**Table 1:** Demographic and clinical characteristics of the study participants.

Variable	Overall (*n* = 89)	Control (*n* = 33)	sPTB (*n* = 32)	iPTB (*n* = 24)	*p*-value
Maternal age, years (IQR)	*N* = 89	30.4 (25.1–34.3)	30.2 (24.6–34.5)	28.3 (22.5–33.3)	0.85
Gestational age at 2nd trimester visit, weeks (IQR)	*N* = 87	16.9 (13.3–18.7)	16.6 (13.7–18.3)	16.7 (14.9–19.3)	0.87
Gestational age at 3rd trimester visit, weeks (IQR)	*N* = 64	33.1 (32.9–34.1)	33.7 (32.3–34.1)	32.7 (32.5–33.2)	0.25
Gestational age at delivery, weeks (IQR)	*N* = 89	39.7 (39.0–40.4)	34.7 (32.3–36.5)	35.9 (33.4–36.2)	**<0.01** ^**a,c**^
BMI, kg/m^2^ (IQR)	*N* = 89	23.7 (21.0–28.3)	27.4 (22.8–33.6)	27.5 (24.2–35.0)	0.06
**Parity**					0.97
0	48%	49%	47%	50%	
≥1	52%	52%	53%	50%	
**Prior preterm birth**					0.27
No prior births	48%	49%	47%	50%	
0	39%	49%	34%	33%	
≥1	12%	3%	19%	17%	
**Smoking history**					0.93
Current smoker	15%	15%	13%	17%	
**BMI categories**					0.49
Underweight (<18.5)	5%	3%	6%	4%	
Normal weight (18.5, 25)	39%	55%	28%	29%	
Overweight (25, 30)	24%	18%	28%	25%	
Obese (30, 40)	26%	21%	25%	33%	
Morbidly obese (≥40)	8%	3%	13%	8%	
**Race/ethnicity**					**0.05** ^**c**^
Black	24%	15%	19%	42%	
Hispanic	23%	18%	31%	17%	
Non-Hispanic White	45%	64%	38%	29%	
Others	9%	3%	13%	13%	

Data are presented as median (inter-quartile range, IQR) or as percentages. *p*-values for between-group comparisons are derived from Kruskal-Wallis test followed by DSCF test for pairwise comparison for continuous variables, and Chi-squared or Fisher’s exact test for categorical variables and are indicated as follows: a - control vs. sPTB; b - sPTB vs. iPTB; c - iPTB vs. control. Percentages may not total 100% due to rounding. Statistically significant differences are bolded.

BMI, body mass index; sPTB, spontaneous preterm birth; iPTB, medically indicated preterm birth; IQR, interquartile range.

### 3.2. TTV and TTMV and timing of delivery

**Figure 3:**
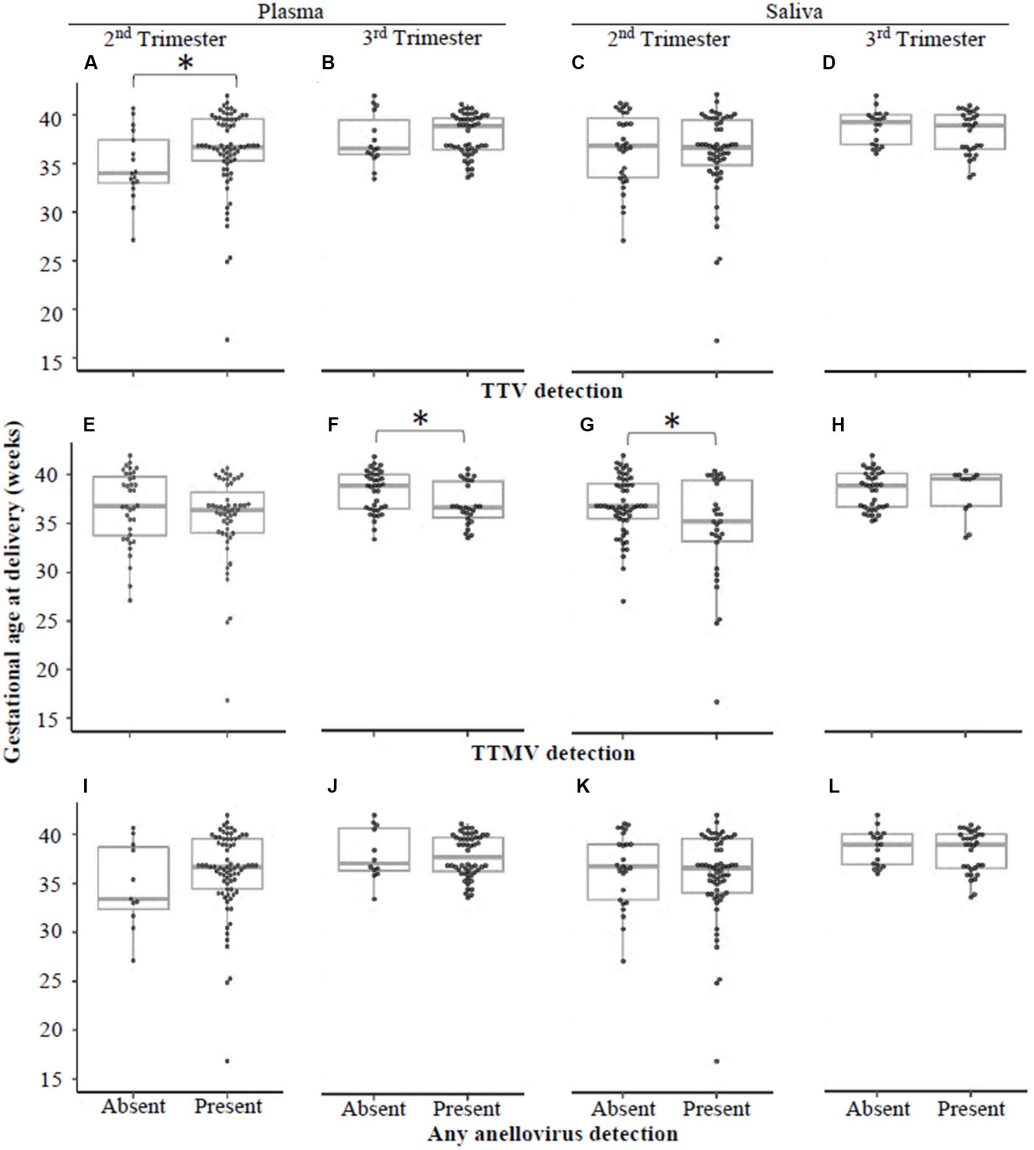
Gestational age at delivery by detection of TTV or TTMV. Samples were stratified by whether they had detectable quantities of TTV **(A–D)**, TTMV **(E-H)**, or any anellovirus **(I–L)**, and gestational ages at delivery (expressed in weeks, with box-and-whiskers plots showing median and interquartile range) were compared between virus-positive and virus-negative groups using Wilcoxon rank sum tests. Statistically significant differences are indicated by an asterisk.

Using qRT-PCR, we tested for the presence of TTV or TTMV DNA in plasma and saliva samples collected at clinic visits in the second and third trimesters. We then stratified the study population by detection of each virus (i.e., virus present or absent) in plasma and saliva at each visit, and tested whether gestational age at delivery differed between those who were virus-positive or virus-negative. Figure 3 shows that detectable TTMV in third trimester plasma and second trimester saliva are both associated with *lower* gestational age at delivery, and that detectable TTV in second trimester plasma is associated with *higher* gestational age at delivery.

**Figure 4:**
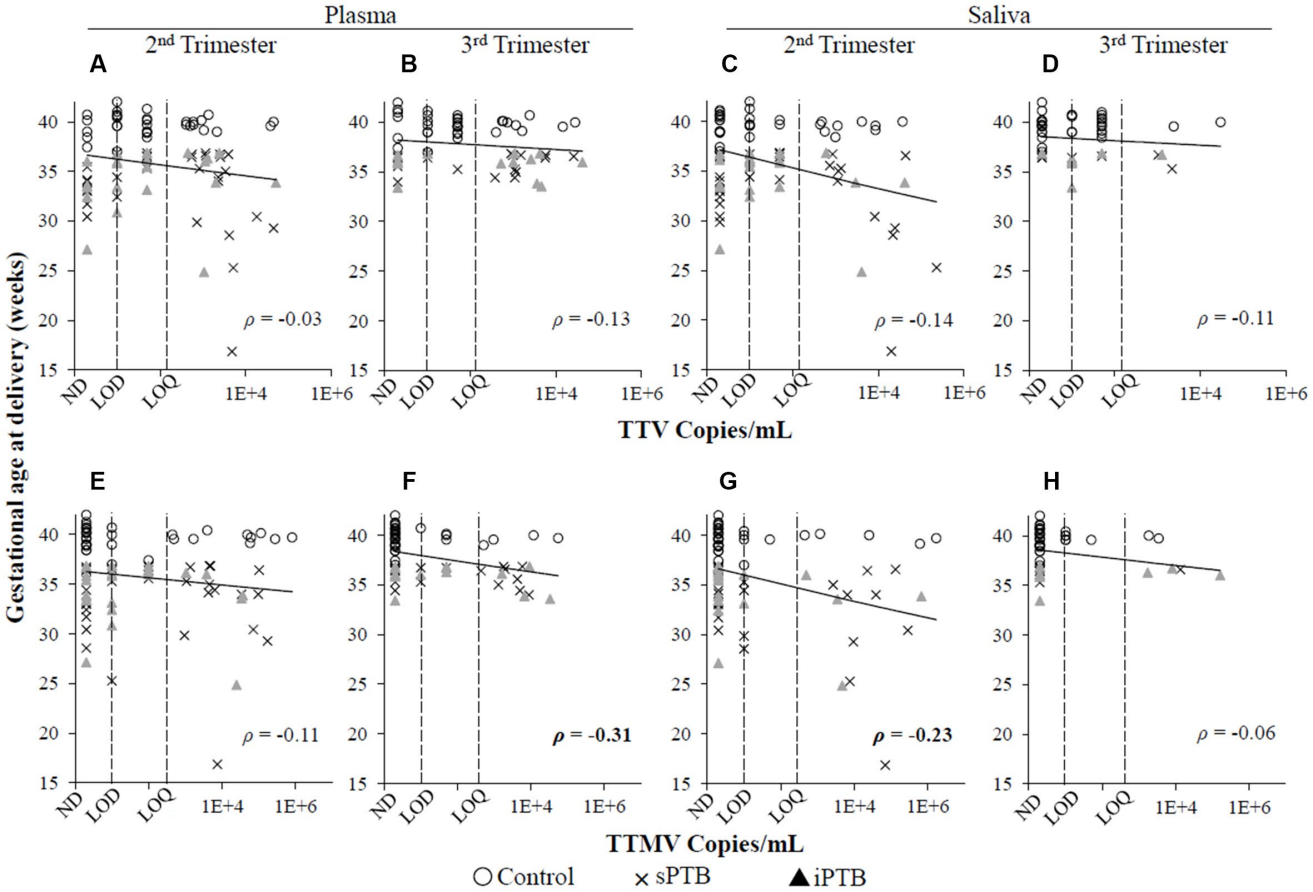
Concentrations of TTV or TTMV and gestational age at delivery. Spearman’s rank correlation coefficients (ρ) between gestational age at delivery and TTV **(A–D)** and TTMV **(E–H)** concentrations in plasma and saliva in the second and third trimesters. 0.20 < |ρ| < 0.29 and 0.30 < |ρ| < 0.39 indicate weak and moderate correlations, respectively. The limits of quantification (LOQ) and detection (LOD) are indicated with dashed lines. Samples without detectable amplification are indicated as not detected (ND); those with amplification in 1 of 2 replicates are plotted at the LOD; those with amplification in 2 of 2 replicates but whose concentrations are below the LOQ are plotted between the LOD and LOQ.

**Figure 5:**
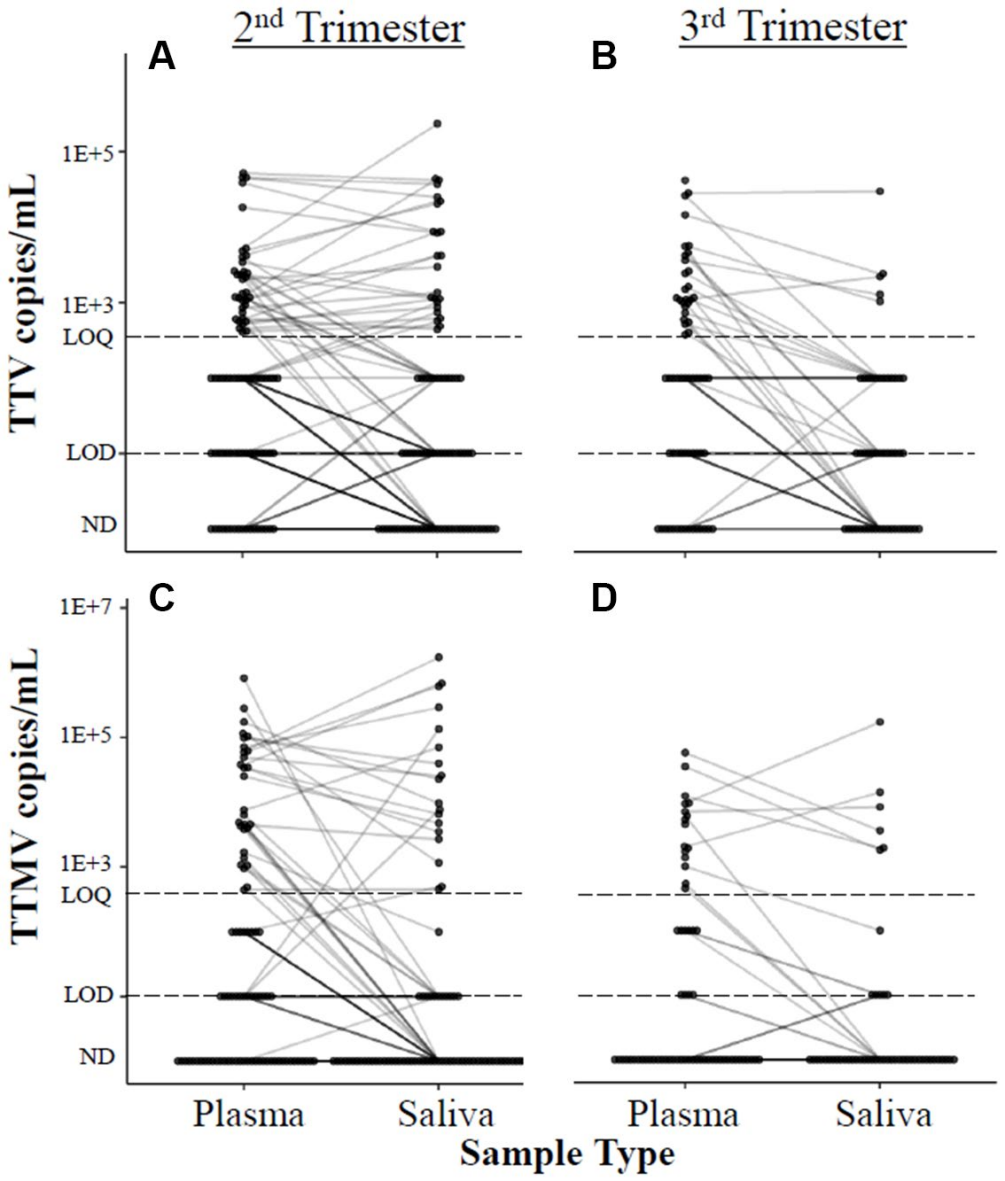
Correlation between anellovirus concentrations in plasma and saliva. Concentrations of TTV **(A,B)** and TTMV **(C,D)** in matched plasma and saliva samples are connected with a line. If no matched sample was available, the unmatched concentration is shown without a connecting line. The limits of quantification (LOQ) and detection (LOD) are indicated with dashed lines. Samples without detectable amplification are indicated as not detected (ND); those with amplification in 1 of 2 replicates are plotted at the LOD; those with amplification in 2 of 2 replicates but whose concentrations are below the LOQ are plotted between the LOD and LOQ. Mean differences between matched samples were assessed using mixed linear models. No significant trends were found.

When the data were analyzed by viral concentration (as opposed to presence/absence of detectable viral particles), only the correlation between timing of delivery and TTMV remained. Gestational age at delivery was moderately (0.30 < |ρ| ≤0.39) negatively correlated with TTMV plasma concentrations in the third trimester (i.e., higher TTMV concentrations associated with earlier delivery) and weakly (0.20 < |ρ| ≤0.29) negatively correlated with salivary TTMV concentrations in the second trimester (Figure 4). Concentrations of TTV and TTMV were not significantly different between sample types (i.e., plasma and saliva) at either visit (Figure 5).

**Figure 6:**
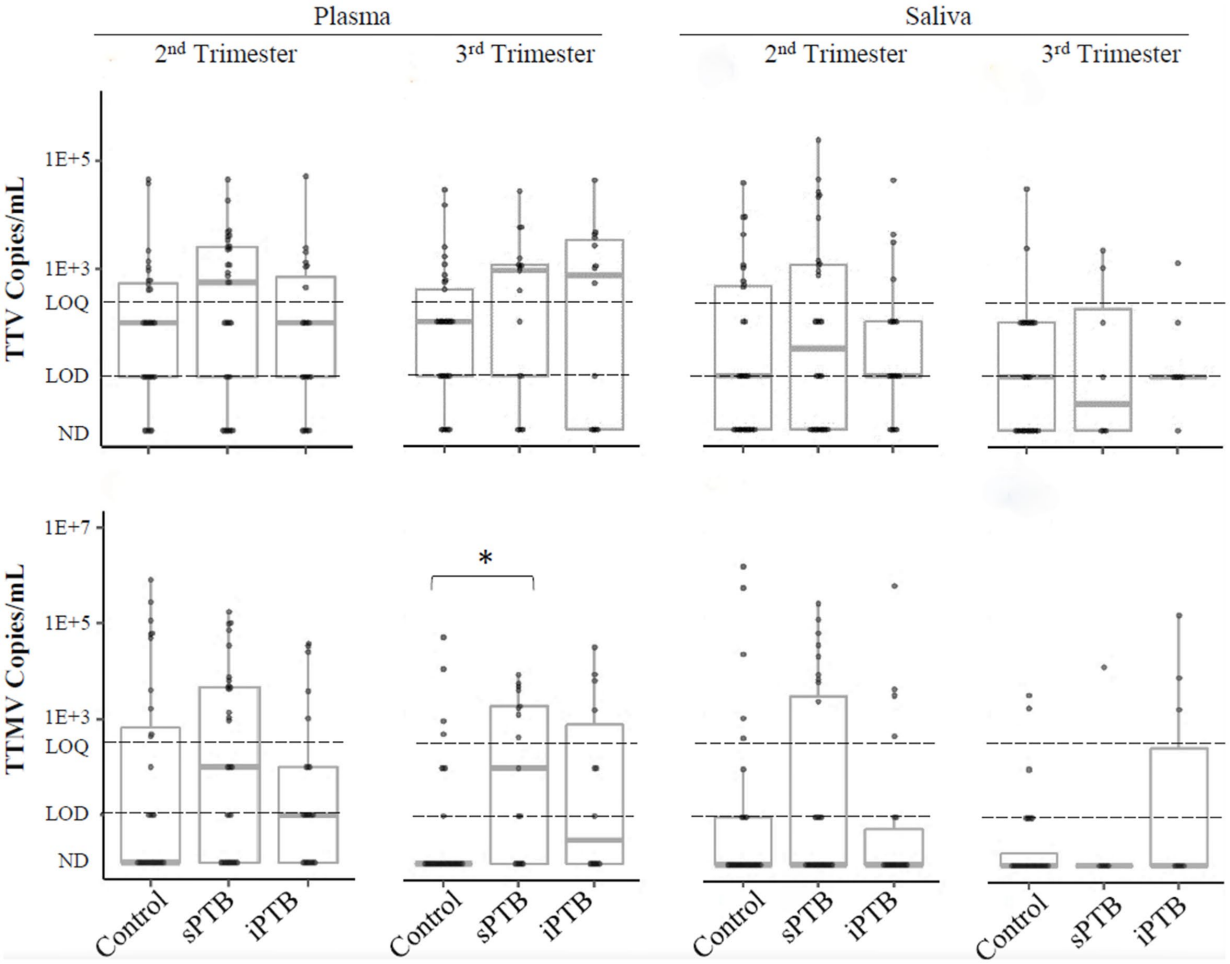
TTV and TTMV load in control, sPTB, and iPTB participants. Viral load is expressed as viral copies/ml of plasma or saliva with box-and-whiskers plots showing median and interquartile range. The limits of quantification (LOQ) and detection (LOD) are indicated with dashed lines. Samples without detectable amplification are indicated as not detected (ND); those with amplification in 1 of 2 replicates are plotted at the LOD; those with amplification in 2 of 2 replicates but whose concentrations are below the LOQ are plotted between the LOD and LOQ. Between-group comparisons were assayed by Kruskal-Wallis tests followed by DSCF. Statistically significant differences are indicated by an asterisk.

We next examined anellovirus detection patterns in term (control) and preterm deliveries that were spontaneous (sPTB) or medically indicated (iPTB). Detection rates of TTV, TTMV, or any anellovirus among these groups are shown in Table 2. TTV prevalence was similar between controls, sPTB, and iPTB participants in both second and third trimesters for both plasma and saliva. However, 65% of sPTB third trimester plasma samples were TTMV-positive, vs. 24% of controls (iPTB, at 50%, was not significantly different from either sPTB or controls). When the data were examined by viral concentrations, a similar pattern was seen: plasma TTMV concentrations were significantly higher in the third trimester among sPTB compared to controls (Figure 6). TTV and TTMV concentrations for iPTB participants were not significantly different from those of either of the other two groups (control and sPTB).

**Table 2:** Prevalence of TTV and TTMV in control, sPTB, and iPTB participants.

	Overall (*n* = 89)	Control (*n* = 33)	sPTB (*n* = 32)	iPTB (*n* = 24)	
	Prevalence, %				*p*-value
**TTV**					
2nd trimester plasma	81	84	78	78	0.78
3rd trimester plasma	77	82	77	64	0.46
2nd trimester saliva	64	56	63	77	0.28
3rd trimester saliva	60	53	50	90	0.09
**TTMV**					
2nd trimester plasma	59	47	66	65	0.24
3rd trimester plasma	41	24	65	50	**0.02** ^**a**^
2nd trimester saliva	35	31	44	27	0.40
3rd trimester saliva	24	25	13	30	0.80
**Any anellovirus**					
2nd trimester plasma	87	88	84	91	0.92
3rd trimester plasma	81	82	88	71	0.53
2nd trimester saliva	71	66	69	82	0.41
3rd trimester saliva	64	59	50	90	0.14

Samples for which Cqs were below the limit of detection (Cqs >40 cycles for TTV or >50 cycles for TTMV), were classified as “virus-negative”; otherwise, they were classified as “virus-positive.” The proportion of virus-positive samples for each birth outcome group are reported. The number of samples for each trimester and sample type per birth outcome group are presented in Figure 1. Statistical significance was calculated using Chi-squared or Fisher’s exact test with pairwise comparisons and are indicated as follows: a - control vs. sPTB; b - sPTB vs. iPTB; c - iPTB vs. control. Statistically significant differences are bolded.

### 3.3. No change in TTV and TTMV load between second and third trimesters

**Figure 7:**
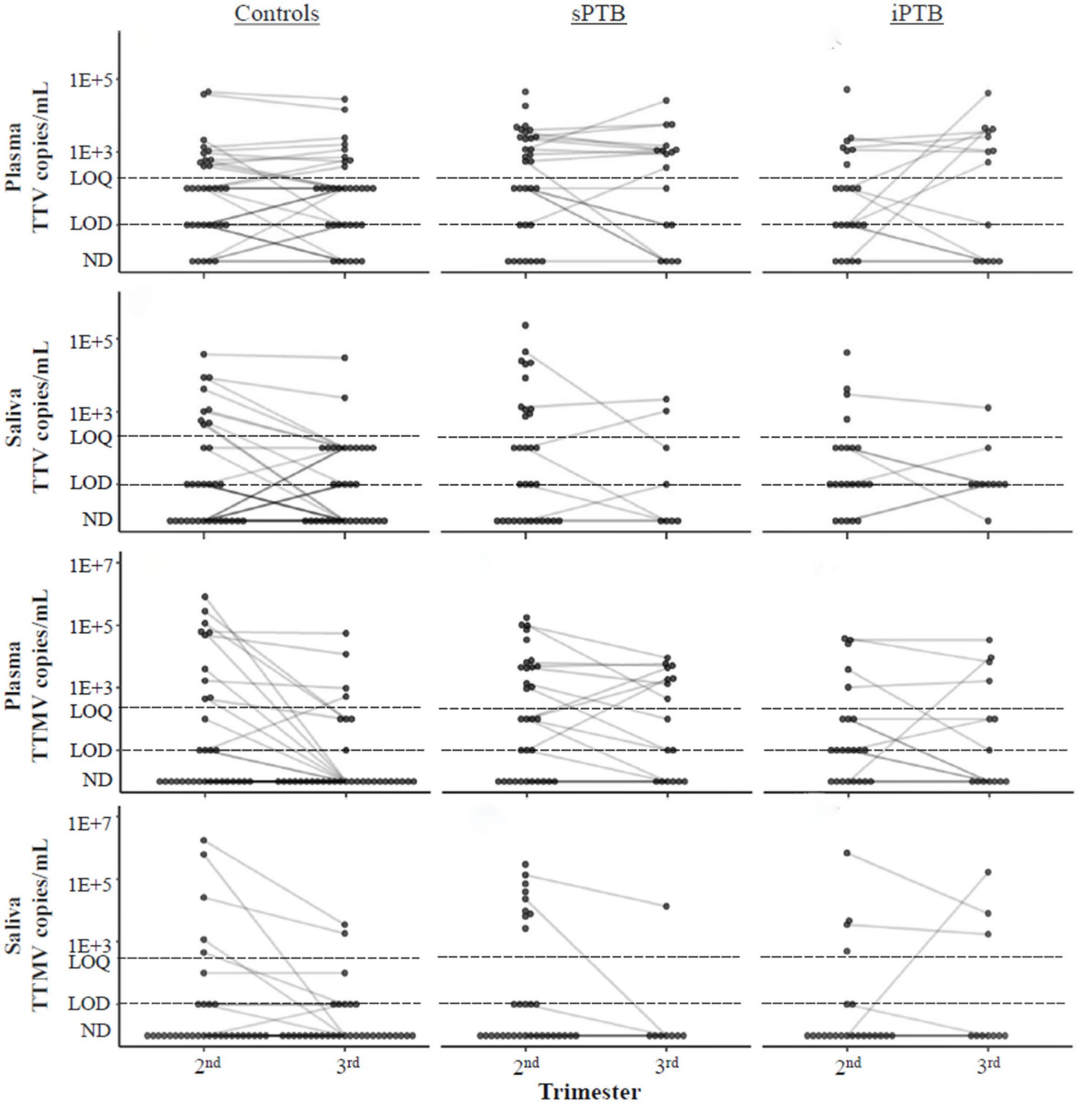
Temporal changes in TTV and TTMV concentrations over the course of pregnancy. Concentrations of TTV and TTMV in plasma and saliva of controls, sPTB, and iPTB groups in the second and third trimesters. Matched samples from the same participant in different trimesters are connected with lines to show change over time. If no matched sample was available, the unmatched concentration is shown without a connecting line. The limits of quantification (LOQ) and detection (LOD) are indicated with dashed lines. Samples without detectable amplification are indicated as not detected (ND); those with amplification in 1 of 2 replicates are plotted at the LOD; those with amplification in 2 of 2 replicates but whose concentrations are below the LOQ are plotted between the LOD and LOQ. Concentrations were compared between the second and third trimesters and within birth outcome groups using mixed linear models. No significant trends were found.

We next examined PCR detection kinetics of TTV and TTMV over the course of gestation (Figure 7). No differences were seen between the second and third trimesters for any of the patient groups in TTV and TTMV viral loads.

### 3.4. Anelloviruses and race/ethnicity

**Figure 8:**
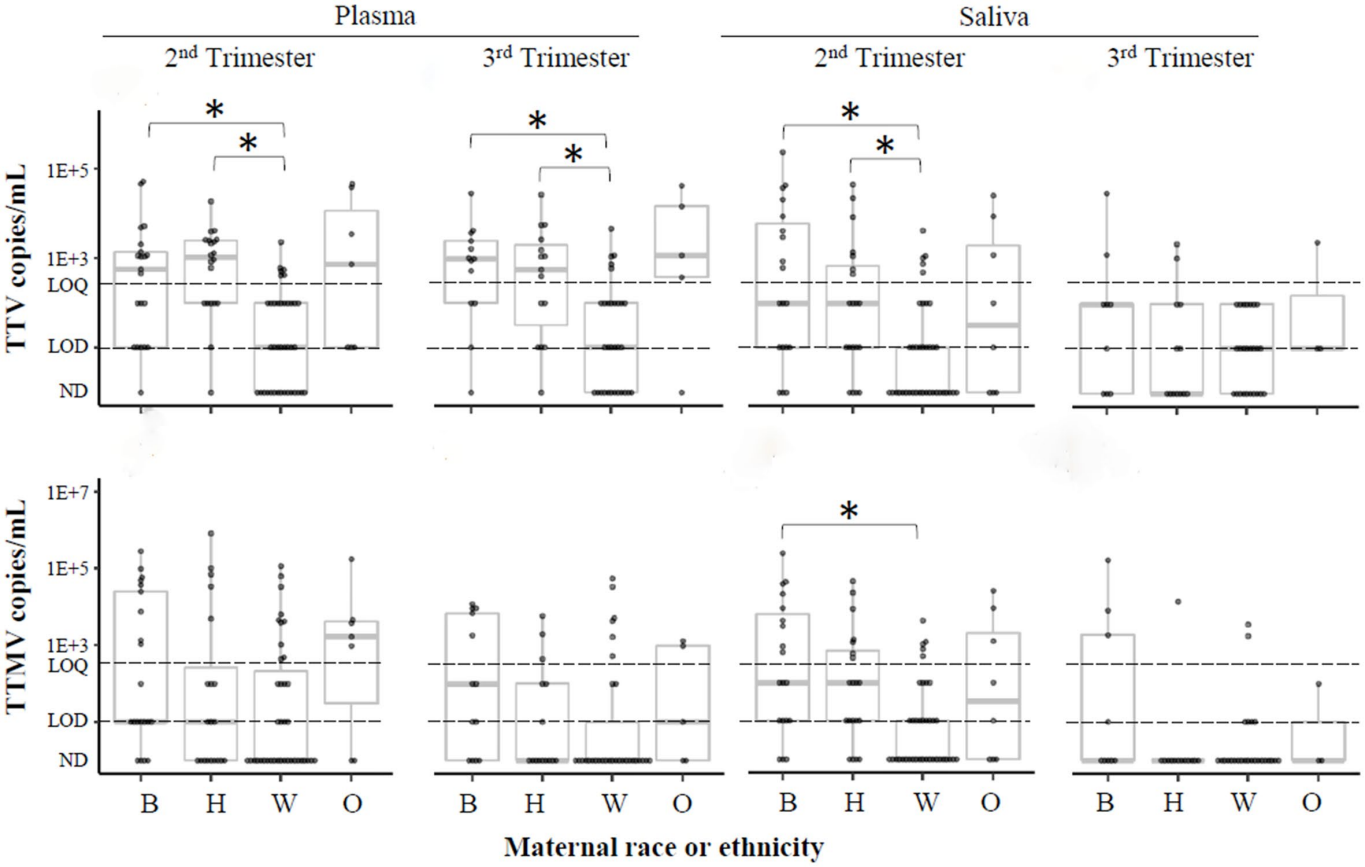
TTV and TTMV load by race and ethnicity. Samples were stratified by participants’ self-reported race or ethnicity, and concentrations of TTV and TTMV were compared between these groups. Concentrations are expressed in copies/mL plasma or saliva with box-and-whiskers plots showing median and interquartile range. The limits of quantification (LOQ) and detection (LOD) are indicated with dashed lines. Samples without detectable amplification are indicated as not detected (ND); those with amplification in 1 of 2 replicates are plotted at the LOD; those with amplification in 2 of 2 replicates but whose concentrations are below the LOQ are plotted between the LOD and LOQ. Between-group comparisons were assayed using Kruskal-Wallis tests followed by DSCF. Statistically significant differences are indicated by an asterisk. B, Black; H, Hispanic; W, non-Hispanic White; O, others.

Black race is associated with PTB (44, 45). A recent study (46) found a link between Black race and anellovirus PCR-positivity in pregnant people. To investigate this correlation in the present cohort, samples were stratified by maternal race and ethnicity, and anellovirus prevalence was compared between these groups (Table 3). While anellovirus prevalence was high in all races and ethnicities, prevalence (of TTV or any anellovirus) was significantly lower in plasma from non-Hispanic White participants compared to Black and Hispanic participants at both study visits, as well as in saliva in the second trimester. A similar pattern was seen when the data were examined using viral concentrations (Figure 8).

**Table 3:** Prevalence of TTV and TTMV by race and ethnicity.

	Overall (*n* = 89)	Black (*n* = 21)	Hispanic (*n* = 20)	Non-Hispanic White (*n* = 40)	Others (*n* = 8)	*p*-value
	Prevalence, %					
**TTV**						
2nd trimester plasma	81	95	95	62	100	**<0.01**
3rd trimester plasma	77	92	93	61	80	**0.05**
2nd trimester saliva	64	84	80	46	63	**0.01**
3rd trimester saliva	60	67	46	60	100	0.44
**TTMV**						
2nd trimester plasma	59	81	55	46	71	0.06
3rd trimester plasma	41	69	40	26	60	**0.04**
2nd trimester saliva	35	63	35	18	50	**<0.01**
3rd trimester saliva	24	44	8	24	33	0.20
**Any anellovirus**						
2nd trimester plasma	87	100	100	72	100	**<0.01**
3rd trimester plasma	81	100	93	68	80	**0.03**
2nd trimester saliva	71	95	80	54	75	**<0.01**
3rd trimester saliva	64	78	46	64	100	0.30

Samples were stratified by participants’ self-reported race and ethnicity, and detection rates of TTV and TTMV were compared between these groups using Chi-squared or Fisher’s exact tests. The number of samples available for each trimester and sample type per birth outcome group are reported in Figure 1. Statistically significant differences are bolded.

In light of these results, we re-evaluated the statistically significant associations described above according to race/ethnicity (Supplementary Table S1). TTMV prevalence in birth outcome groups was not significantly different (except among Hispanic participants, but pairwise follow-up tests (sPTB vs. control, sPTB vs. iPTB, iPTB vs. control) were not significant); however, it should be noted that the stratified analysis was limited by small numbers of subjects in each group.

### 3.5. Anelloviruses and parity

**Figure 9:**
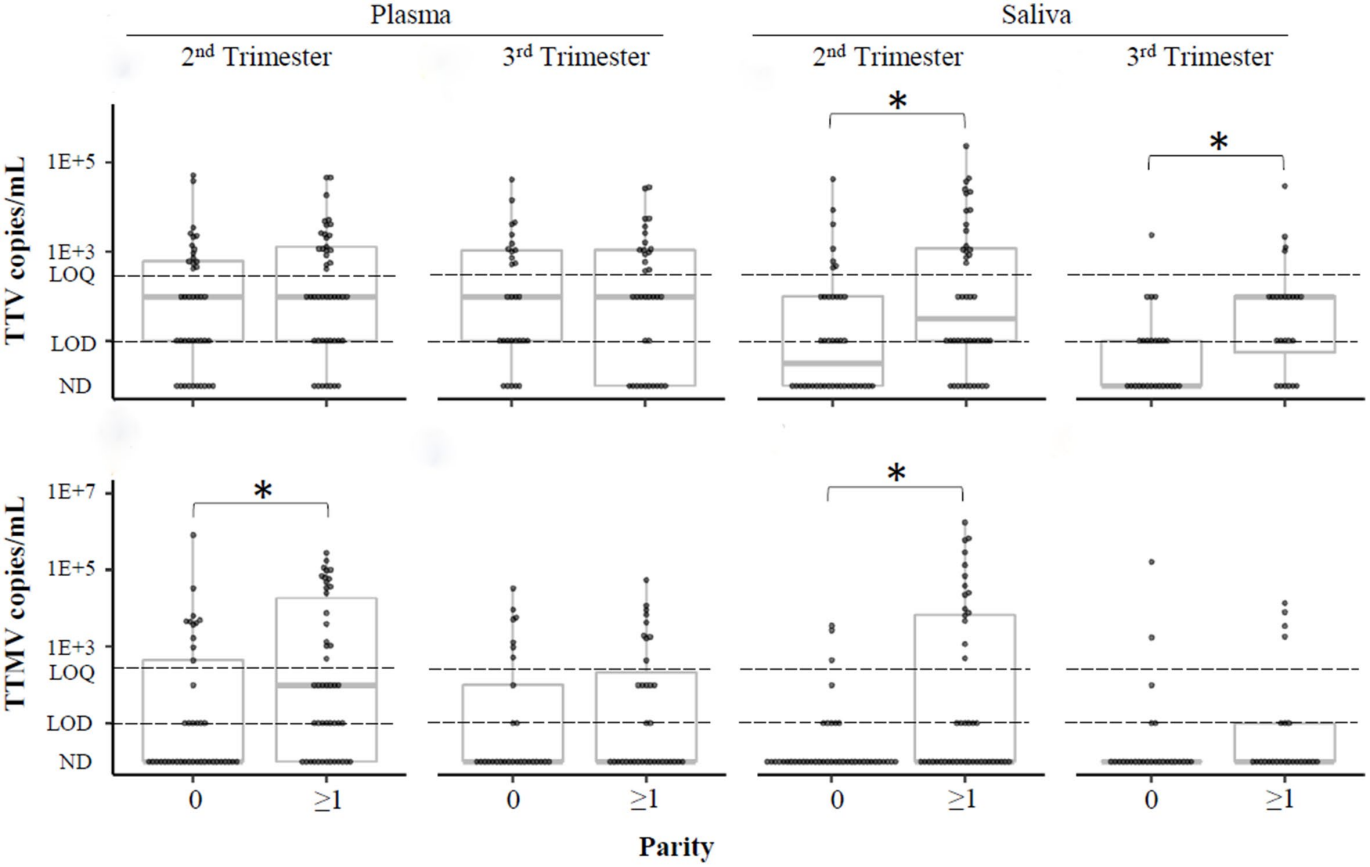
TTV and TTMV load in nulliparous and parous pregnant participants. Samples were stratified by whether participants had previously given birth (parity ≥1) or not (parity =0) and concentrations of TTV and TTMV were compared between these groups. Concentrations are expressed in copies/mL with box-and-whiskers plots showing median and interquartile range. The limits of quantification (LOQ) and detection (LOD) are indicated with dashed lines. Samples without detectable amplification are indicated as not detected (ND); those with amplification in 1 of 2 replicates are plotted at the LOD; those with amplification in 2 of 2 replicates but whose concentrations are below the LOQ are plotted between the LOD and LOQ. Between-group comparisons were assayed using Wilcoxon rank sum tests. Statistically significant differences are indicated by an asterisk.

To understand the association between parity and anellovirus prevalence, we categorized the pregnant population into two groups: individuals who had never given birth (“nulliparous,” parity =0), and those with at least 1 prior birth (“parous,” parity ≥1). Differences in TTV and TTMV viral concentrations and prevalence between nulliparous and parous participants are shown in Figure 9 and Supplementary Table S2. TTV was more prevalent in saliva in parous than in nulliparous participants, and TTMV was more prevalent in both plasma and saliva in parous participants in the second trimester. Similar findings were present when the data were analyzed by concentration.

### 3.6. No association of maternal BMI with anellovirus detection

Given that obesity increases the risk of preterm delivery (47), we compared rates of TTV and TTMV detection in plasma and saliva specimens in normal, overweight, and obese participants. Supplementary Table S3 indicates no statistically significant differences in anellovirus detection between BMI categories.

### 3.7. Anellovirus detection and maternal history of PTB

People who delivered preterm in a previous pregnancy are at risk of preterm delivery in a subsequent pregnancy, but the etiology of recurrent PTB is unclear (48). We therefore compared the prevalence of TTV and TTMV in parous participants with no history of PTB (*n* = 35) or ≥ 1 prior PTB (*n* = 12), shown in Supplementary Table S4. The prevalence of TTV and TTMV were comparable between groups.

## 4. Discussion

### 4.1. Anelloviruses are highly prevalent in biological samples across gestation

This study investigated the prevalence of the anellovirus subtypes TTV (from genus *Alphatorquevirus*) and TTMV (*Betatorquevirus*) in pregnant populations and their association with preterm delivery (medically indicated and spontaneous). TTV, the prototypical anellovirus, was detected in ≥75% of plasma samples and ≥60% of saliva samples (Table 2), with little change between the second and third trimesters (Figure 7). TTMV was identified in ≥40% of plasma samples and ≥20% of saliva samples (Table 2), with no significant changes in TTMV concentration between study visits (Figure 7). These high rates of TTV and TTMV prevalence are consistent with other reports in the literature (20, 49, 50).

### 4.2. Anelloviruses and birth outcomes

In this study population, second trimester saliva and third trimester plasma TTMV were associated with sPTB and shorter length of gestation (Table 2, Figures 3, 4, 6). These findings are consistent with those of our previous publication (28), in which TTMV was more commonly found in serum from participants laboring in the preterm period than in participants laboring at term.

These results might be interpreted in at least two ways: first, that TTMV has a role in the onset of preterm labor. Such a phenomenon might be mediated through a direct effect of TTMV or indirectly, through interactions between TTMV and other microbes or non-infectious inflammatory processes that regulate parturition. A second interpretation of our results is that TTMV has no role in parturition, but nonetheless serves as a marker for sPTB in pregnancy (i.e., that an independent factor(s) influences both duration of pregnancy and TTMV load). Substantial evidence links anellovirus load with immune status, with higher loads suggestive of immunosuppression (51). People receiving a solid organ transplant (52, 53), and those with cancer (27, 54), HIV infection (55), and sepsis (56) have higher plasma anellovirus concentrations than healthy controls. Healthy pregnancy is associated with an elevation in the immune suppressive CD25+ CD4+ regulatory T cell subset (57). These regulatory cells suppress antigen-specific immune responses that are important for allograft tolerance and for sustaining pregnancy to full term (58, 59). Anellovirus replication is believed to occur in activated T lymphocytes (60). Given that immune system activation in plasma is at its lowest level in healthy pregnancy (61–63), alterations in immune status that lead to pathological early delivery might simultaneously increase TTMV load.

The association of TTV detection with the timing of parturition is less clear. In our prior report (28), TTV positivity was associated with preterm delivery, a finding that was not replicated in the present study. Here, we report that TTV-positivity in plasma (but not saliva) in the second trimester is associated with higher gestational age at delivery (Figure 3). This finding was not sustained when the data were examined by viral concentration (Figures 3, 4) or by patient group (control, sPTB, or iPTB, Table 2, Figure 6). The differences between our prior report and the current one have been noted above and include: in the prior study, all samples were collected from participants in labor (whether term or preterm) and were assayed using non-quantitative PCR methodology with substantially lower sensitivity than the assays used in the current study. The present findings are consistent with Sloan et al. (49), who found no correlation between TTV prevalence or viral load in maternal blood and spontaneous preterm labor.

We also found that anellovirus concentrations are largely stable across gestation (Figure 7), which complement findings by Stout et al. (46), who observed similar anellovirus concentrations during each trimester and at delivery.

### 4.3. Association of anelloviruses with other demographic variables

#### 4.3.1. Race and ethnicity

Race is associated with preterm birth (44, 45). In our study cohort, 24% of the participants were Black. These participants had higher rates of detection and concentrations of TTV, TTMV, or any anellovirus compared to non-Hispanic White individuals (Table 3, Figure 8). This is consistent with Stout et al. (46), who observed racial differences in anellovirus positivity in Black compared to White pregnant individuals. We also report that anellovirus prevalence and load in Hispanic participants was similar to that of Black participants, and greater than that of non-Hispanic White participants (Figure 8), a novel finding.

Race and ethnicity designations were not evenly distributed in our population, with non-Hispanic White participants representing 64% of controls, 38% of sPTB, and 29% of iPTB, Table 1. It is possible that an interaction between race/ethnicity and anellovirus positivity accounts for the differential occurrence of lower gestational age at birth according to the presence of anellovirus (Table 2, Figure 3). We examined this hypothesis using stratified analysis by race and ethnicity, but no clear patterns emerged (Supplementary Table S1). This finding should be interpreted with caution because of the small numbers of subjects in the stratified analysis. This possibility of interaction with race and ethnicity is an important consideration for future studies.

#### 4.3.2. Parity

In this analysis, we report an association between TTV and TTMV detection and increasing parity (no prior births vs. ≥1 prior birth) (Figure 9), which corroborates our past observation (28). Several hypotheses might explain why parous individuals have higher anellovirus loads than nulliparous individuals: (1) It may be a function of age (64–67). However, anellovirus concentrations generally remain stable between the ages of 20 and 50 (64, 65, 67), making this explanation less likely. (2) Vertical transmission might occur from offspring to parent. By 3 months of age, about half of the infants studied by Bagaglio et al. (68) harbored unique anellovirus species not detected at delivery in their birthing parent. (3) New anelloviruses might be acquired during each pregnancy, possibly related to the immune modifications discussed above. Parous individuals may have higher background inflammation (as measured by cytokine profiles (62, 69, 70) and microbiota composition (71)) than nulliparous individuals, and thus may be more susceptible to anellovirus colonization (25, 26).

#### 4.3.3. Maternal BMI

There is evidence that maternal obesity increases the risk of PTB (72, 73), and that TTV load is higher in obese compared to normal weight individuals (74). In the present study (Supplementary Table S3) we found no significant association between anellovirus detection and maternal BMI; however, such associations might be found in a future study with a larger cohort.

#### 4.3.4. History of PTB

No association was found between anellovirus load and prior PTB (a known risk factor for preterm delivery in subsequent pregnancy (48), Supplementary Table S4). These results need to be interpreted with caution due to the small sample size.

### 4.4. Strengths and limitations

Strengths of this case–control study include the following: (1) samples were obtained prospectively in monitored pregnancies; (2) we included two relevant comparison groups (controls delivering at term and those who had medically indicated PTB); (3) the population is diverse; (4) the multi-center design enhances the likelihood of generalizability; (5) we developed highly sensitive PCR assays (lower limits of quantitation ~300 copies of TTV/mL and ~400 copies of TTMV/mL).

This study has several limitations. First, 48% of participants had one or more missing samples (Figure 1). This had a potential negative impact on power, especially in analyses of third trimester data, by which time 11 participants had already delivered prematurely. The presence of missing data and small sample size together is likely to have hampered our ability to investigate the association between TTMV positivity and sPTB by race and ethnicity. Second, for some of our quantitative analyses, it was necessary to assign arbitrary values to samples that had either (a) detectable amplification that fell below the lower limit of the standard curves, or (b) one detectable and one non-detectable value. In doing so, we followed published methods (40, 41, 75, 76).

Though it is not a limitation *per se*, we did not confirm results of prior reports that TTV has tropism to saliva (23), where it has been reported in concentrations 100–1,000 times higher than corresponding serum samples (23, 77, 78). In the present study, viral concentrations were comparable between plasma and saliva samples (Figure 5). Our findings may have been influenced by differences in collection techniques (i.e., with a swab in the current study and by expectoration in other studies) (23, 79–84), nucleic acid extraction (23, 79, 80, 82, 85), and PCR primers/assay procedures (72, 78–80).

Substantial evidence suggests that variations in anellovirus load are associated with the microbial milieu of specific compartments (22, 39). However, we did not explore these relationships.

### 4.5. Conclusion

Anelloviruses remain an intriguing, but as yet poorly understood, variable in relation to the timing of parturition. In this study, second and third trimester TTMV was associated with lower gestational age at delivery and spontaneous preterm birth. TTV showed no correlation with spontaneous preterm birth. Both TTV and TTMV prevalence and viral load varied significantly by participants’ race and ethnicity, which may have impacted results relating anelloviruses and birth outcomes. Further investigation into TTMV as a potential circulating biomarker of preterm birth is warranted, as well as experiments to clarify whether anelloviruses have a causal effect on the timing of parturition, or are bystanders affected by underlying processes that independently govern gestational length.

## Data availability statement

The original contributions presented in the study are included in the article/Supplementary material, further inquiries can be directed to the corresponding author.

## Ethics statement

The studies involving human participants were reviewed and approved by Institutional Review Board of Northwestern University. The patients/participants provided their written informed consent to participate in this study.

## Author contributions

CK, MS, and CR carried out the experiments. MS organized the data. CK wrote the original draft. LS performed the statistical analyses and contributed to the original draft. CK, MS, and EH interpreted the data, reviewed and edited the manuscript. CK and EH conceptualized the project, determined its methodology, acquired funding and resources. LK-D and AB provided biological samples for the study. All authors contributed to the article and approved the submitted version.

## Funding

This work was supported by the NICHD R01 grant to EH (R01HD096209) and NorthShore University HealthSystem Pilot Grant to CK (EH21-115). Financial support for the MOMS study was provided by the following sources: HHSN275201200007I-HHSN27500005 of the National Children’s Study: Vanguard Study – Task Order 5: Stress and Cortisol Measurement for the National Children’s Study [Principal Investigator: AB (MD, MSc, MPH)].

## Conflict of interest

The authors declare that the research was conducted in the absence of any commercial or financial relationships that could be construed as a potential conflict of interest.

## Publisher’s note

All claims expressed in this article are solely those of the authors and do not necessarily represent those of their affiliated organizations, or those of the publisher, the editors and the reviewers. Any product that may be evaluated in this article, or claim that may be made by its manufacturer, is not guaranteed or endorsed by the publisher.

## Abbreviations

iPTB: Indicated preterm birth
MOMS: Measurement of maternal stress
sPTB: Spontaneous preterm birth
TTV: Torque teno virus
TTMV: Torque teno mini virus
