# Supplementary material for: Maternal plasma and salivary anelloviruses in pregnancy and preterm birth
Source: Front Med (Lausanne). 2023 Jun 15;10:1191938. doi: 10.3389/fmed.2023.1191938 (PMC10309558; doi:10.3389/fmed.2023.1191938)
Supplement: Supplementary file 1 [file Table_1.DOCX]

## Supplementary Table 1. Prevalence of TTMV in third trimester plasma by birth outcome group and race/ethnicity.

This is a follow-up analysis of significant associations reported in **Table 2**, conducted to investigate the interaction between race/ethnicity and TTMV detection in birth outcome groups (sPTB, iPTB, or control). Samples were stratified by birth outcome group and maternal race or ethnicity, and detection rates of TTV and TTMV were compared between these groups. Between-group comparisons were assayed using Chi-squared or Fisher’s exact tests.

|  | **Overall**  (n=64) | **Control**  (n=33) | **sPTB**  (n = 17) | **iPTB**  (n=14) |  |
| --- | --- | --- | --- | --- | --- |
|  | Prevalence, % (N) | | | | p-value |
| **TTMV, overall** |  |  |  |  |  |
| 3^rd^ trimester plasma | 41% (26) | 24% (8) | 65% (11) | 50% (7) | 0.02 |
| **TTMV, stratified by race/ethnicity** | | | | |  |
| Black | 69% (9) | 60% (3) | 100% (4) | 50% (2) | 0.44 |
| Hispanic | 40% (6) | 0% (0) | 67% (4) | 67% (2) | 0.05 |
| Non-Hispanic White | 26% (8) | 19% (4) | 40% (2) | 40% (2) | 0.57 |
| Others | 60% (3) | 100% (1) | 50% (1) | 50% (1) | 0.66 |
| Abbreviations: TTMV, torque teno midi virus; sPTB, spontaneous preterm birth; iPTB, medically indicated preterm birth | | | | | |
