# Supplementary material for: Maternal plasma and salivary anelloviruses in pregnancy and preterm birth
Source: Front Med (Lausanne). 2023 Jun 15;10:1191938. doi: 10.3389/fmed.2023.1191938 (PMC10309558; doi:10.3389/fmed.2023.1191938)
Supplement: Supplementary file 2 [file Table_2.DOCX]

## Supplementary Table 2. Prevalence of TTV and TTMV in nulliparous and parous pregnant participants.

Samples were stratified by whether participants had previously given birth (parity ≥1) or not (parity =0), and detection rates of TTV and TTMV were compared between these groups. The number of samples for each trimester and sample type per birth outcome group are reported in Figure 1. Between-group comparisons were assayed using Chi-squared or Fisher’s exact tests.

|  | **Overall**  (n=89) | **Parity** | |  |
| --- | --- | --- | --- | --- |
|  |  | **0**  (n=43) | **≥1**  (n=46) |  |
|  | Prevalence, % | | | p-value |
| **TTV** |  |  |  |  |
| 2^nd^ trimester plasma | 81 | 76 | 85 | 0.28 |
| 3^rd^ trimester plasma | 77 | 83 | 71 | 0.29 |
| 2^nd^ trimester saliva | 64 | 50 | 77 | **0.01** |
| 3^rd^ trimester saliva | 60 | 46 | 75 | **0.04** |
| **TTMV** |  |  |  |  |
| 2^nd^ trimester plasma | 59 | 44 | 72 | **0.01** |
| 3^rd^ trimester plasma | 41 | 35 | 46 | 0.36 |
| 2^nd^ trimester saliva | 35 | 21 | 48 | **0.01** |
| 3^rd^ trimester saliva | 24 | 19 | 29 | 0.41 |
| **Any anellovirus** |  |  |  |  |
| 2^nd^ trimester plasma | 87 | 81 | 94 | 0.07 |
| 3^rd^ trimester plasma | 81 | 83 | 80 | 0.78 |
| 2^nd^ trimester saliva | 71 | 57 | 84 | **0.01** |
| 3^rd^ trimester saliva | 64 | 50 | 79 | **0.03** |
