# Supplementary material for: Maternal plasma and salivary anelloviruses in pregnancy and preterm birth
Source: Front Med (Lausanne). 2023 Jun 15;10:1191938. doi: 10.3389/fmed.2023.1191938 (PMC10309558; doi:10.3389/fmed.2023.1191938)
Supplement: Supplementary file 3 [file Table_3.DOCX]

## Supplementary Table 3. Prevalence of TTV and TTMV in plasma and saliva from normal, overweight, and obese participants.

Participants with body mass indices (BMI, kg/m^2^) <25 were classified as “normal,” those with 25≤ BMI <30, “overweight,” and those with BMI ≥30, “obese.” The number of samples for each trimester and sample type per birth outcome group are reported in Figure 1. Between-group comparisons were assayed using Chi-squared or Fisher’s exact tests.

|  | **Overall**  (n=89) | **Normal** (n=38) | **Overweight** (n=21) | **Obese**  (n=30) |  |
| --- | --- | --- | --- | --- | --- |
|  | Prevalence, % | | | | p-value |
| **TTV** |  |  |  |  |  |
| 2^nd^ trimester plasma | 81 | 78 | 80 | 83 | 0.88 |
| 3^rd^ trimester plasma | 77 | 75 | 73 | 82 | 0.80 |
| 2^nd^ trimester saliva | 64 | 62 | 67 | 64 | 0.94 |
| 3^rd^ trimester saliva | 60 | 55 | 50 | 77 | 0.34 |
| **TTMV** |  |  |  |  |  |
| 2^nd^ trimester plasma | 59 | 57 | 65 | 57 | 0.80 |
| 3^rd^ trimester plasma | 41 | 28 | 47 | 59 | 0.10 |
| 2^nd^ trimester saliva | 35 | 38 | 33 | 32 | 0.88 |
| 3^rd^ trimester saliva | 24 | 24 | 13 | 31 | 0.64 |
| **Any anellovirus** |  |  |  |  |  |
| 2^nd^ trimester plasma | 87 | 89 | 85 | 87 | 0.89 |
| 3^rd^ trimester plasma | 81 | 78 | 80 | 88 | 0.68 |
| 2^nd^ trimester saliva | 71 | 73 | 71 | 68 | 0.90 |
| 3^rd^ trimester saliva | 64 | 59 | 50 | 85 | 0.18 |
