# Supplementary material for: Maternal plasma and salivary anelloviruses in pregnancy and preterm birth
Source: Front Med (Lausanne). 2023 Jun 15;10:1191938. doi: 10.3389/fmed.2023.1191938 (PMC10309558; doi:10.3389/fmed.2023.1191938)
Supplement: Supplementary file 4 [file Table_4.DOCX]

## Supplementary Table 4. Prevalence of TTV and TTMV in pregnant individuals with or without a history of PTB.

The number of samples for each trimester and sample type per birth outcome group are reported in Figure 1. P-values were calculated using Chi-squared or Fisher’s exact tests. Abbreviations: PTB, preterm birth

|  | **All parity ≥1**  (n=46) | **No prior PTB** (n=35) | **Prior PTB** (n=11) |  |
| --- | --- | --- | --- | --- |
|  | Prevalence, % | | | p-value |
| **TTV** |  |  |  |  |
| 2^nd^ trimester plasma | 85 | 86 | 82 | 0.75 |
| 3^rd^ trimester plasma | 71 | 72 | 67 | 0.78 |
| 2^nd^ trimester saliva | 77 | 73 | 91 | 0.21 |
| 3^rd^ trimester saliva | 75 | 73 | 100 | 0.39 |
| **TTMV** |  |  |  |  |
| 2^nd^ trimester plasma | 72 | 66 | 91 | 0.11 |
| 3^rd^ trimester plasma | 46 | 45 | 50 | 0.82 |
| 2^nd^ trimester saliva | 48 | 46 | 55 | 0.60 |
| 3^rd^ trimester saliva | 29 | 27 | 50 | 0.50 |
| **Any anellovirus** |  |  |  |  |
| 2^nd^ trimester plasma | 94 | 91 | 100 | 0.32 |
| 3^rd^ trimester plasma | 80 | 79 | 83 | 0.82 |
| 2^nd^ trimester saliva | 84 | 82 | 91 | 0.48 |
| 3^rd^ trimester saliva | 79 | 77 | 100 | 0.45 |
